# Listening to music during intranasal (es)ketamine therapy in patients with treatment-resistant depression correlates with better tolerability and reduced anxiety

**DOI:** 10.3389/fpsyt.2024.1327598

**Published:** 2024-01-23

**Authors:** Johannes Hauser, Jan Sarlon, Timur Liwinski, Annette B. Brühl, Undine E. Lang

**Affiliations:** ^1^University of Basel, Basel, Switzerland; ^2^University Psychiatric Clinics (UPK), Basel, Switzerland

**Keywords:** intranasal (es)ketamine therapy, music, TRD (treatment-resistant depression), dissociation, anxiety, dose tolerability

## Abstract

**Background:**

Although the effectiveness of (es)ketamine for therapy-resistant depression (TRD) has been established, potential treatment-limiting factors include side effects like dissociation, anxiety, or elevated blood pressure. Music can reduce stress and negative emotions as anxiety. This study aimed to investigate the impact of listening to music during intranasal (es)ketamine administration on both tolerability and efficacy.

**Methods:**

Records of 494 sessions (of 37 patients) with intranasal (es)ketamine administration, each containing data of blood pressure measurements, DSS-IV (dissociation symptoms scale-IV), anxiety and euphoria analogue scale, MADRS (Montgomery–Åsberg Depression Rating Scale) and BDI (Beck’s Depression Inventory) were evaluated.

**Results:**

The between-group analysis, comparing participants who listened to music with those who did not, revealed significant differences in the administered dose (*p*-value: 0.003, mean: 131.5 mg with music vs. 116.7 mg without music), scores on the DSS Item 1 (*p*-value: 0.005, mean: 3 points vs. 2.4 points), levels of anxiety (*p*-value: <0.001, mean: 0.4 points vs. 1.4 points), and measurements of maximal systolic blood pressure after administration (*p*-value: 0.017, mean: 137.9 mmHg vs. 140.3 mmHg). Listening to music had no impact on the MARDS-change score between the sessions.

**Limitations:**

Key limitations include a non-randomized naturalistic design and the non-standardized selection of music, which was based on individual patient preferences.

**Conclusion:**

Listening to music during intranasal (es)ketamine therapy appears to be linked to reduced anxiety and lower blood pressure, stable or increased dissociation levels, and improved tolerance for higher doses. These findings could potentially contribute to the optimization of (es)ketamine therapy, both in terms of treatment efficacy and managing side effects.

## Introduction

The use of (es)ketamine or racemic ketamine medication shows promise as a treatment option for both unipolar depression and depressive episodes of bipolar affective disorder. Its effectiveness has been substantiated through multiple controlled trials and meta-analyses (1–3). Ketamine is a racemic mixture composed of equal amounts of (S)-ketamine and (R)-ketamine (4). In this study, the term “(es)ketamine” is used when referring to both racemic ketamine and esketamine. Otherwise, the terms “esketamine” or “racemic ketamine” are used.

Esketamine nasal spray has been approved for the treatment of treatment-resistant depression (TRD) in the United States and Europe since 2019. There is evidence that both response rate and adverse effects are dose-related (5–7), however, the individual dose range in which the antidepressant responds is rather high (8) and individual patient factors play an important role in tolerability and efficacy (9). While (es)ketamine is generally regarded as relatively safe, it is important to note that some side effects have been reported, although they are typically transient. These side effects may include dizziness, blurred vision, headache, dissociation (disruption of and/or discontinuity in the normal integration of consciousness, memory, identity, emotion, perception, body representation, motor control, and behavior) (10), anxiety, restlessness, as well as elevations in blood pressure and heart rate (2, 11, 12). In a multicentric randomized control study comparing esketamine nasal spray plus antidepressant and antidepressant plus placebo, 7% of the intervention group discontinued study drug because of an adverse event, compared to 0.9% in the control group (13). One of the most important and potential treatment-limiting side effects of (es)ketamine are dissociations (14–16).

As both esketamine and racemic ketamine were administered, the differences in efficacy and adverse effects should be considered. There is limited literature directly comparing the two intranasal formulations. However, a recent systematic review of different routes of administration, converting the respective doses, found racemic ketamine to be more effective than esketamine in terms of depression severity, response and remission rates (17). These findings are supported by another previous systematic review (18), comparing intravenous racemic ketamine with intranasal esketamine. One observational study found that fewer treatments were required to achieve remission with intravenous ketamine than with intranasal esketamine (19). Whether separating ketamine from its dissociative effects is of therapeutic benefit to TRD patients remains to be determined (20). There is also no clear evidence of variance in adverse events between ketamine formulations, but all formulations had similar or lower dropout rates, suggesting similar acceptability (17, 18).

There are first case reports in literature showing that listening to music as a nonpharmacologic intervention can reduce dissociation (21) or improve the tolerance to them (15, 22). In clinical populations not primary based on psychiatric diagnoses, listening to music may have a beneficial effect on anxiety (23, 24) or reduce blood pressure (25). But also in patients with depressive disorders, music therapy given in addition to the usual can improve depressive symptoms and shows efficacy in decreasing anxiety levels (26).

Moreover, there are findings of augmentative effects of racemic ketamine and psychotherapy (27, 28). Listening to music can stimulate neurogenesis and neuroplasticity, enhance brain recovery, and normalize stress response (29). Blum et al. hypothesize that music interventions enhance brain white matter plasticity through dopaminergic recruitment (30). Recent findings suggest that music plays an important role in facilitating positive clinical outcomes of psychedelic therapy (31–33). Also in a study on healthy adults receiving lysergic-acid diethylamide (LSD), the effects of LSD on brain entropy was greatest when listening to music (34). Furthermore, Muscat et al. suggest that music is an important component of the therapeutic milieu that helps maintain a positive emotional tone during therapy with ketamine (20). Altogether, when receiving (es)ketamine, listening to music might be beneficial in reducing side effects or increasing primary outcome (depression severity).

At the University Psychiatric Clinics Basel (UPK Basel), therapy with repeated (es)ketamine is used for the treatment of unipolar and bipolar major depression.

To our knowledge, there is no data to the direct comparison of the two settings (intranasal (es)ketamine with music vs. (es)ketamine only) concerning efficacy and side effects. There is one ongoing study – a randomized, single-blind trial with 20 participants and intravenous (es)ketamine (35), however the results have not yet been published.

The objective of this study was to analyze the present patient data (therapy setting, applicated dose, physiological and psychometric data) collected during ambulatory therapy with (es)ketamine in patients with depression. Analysing those data allowed us to compare different forms of therapy setting regarding their efficacy or reduction of side effects. Specifically, the setting in which patients prefer to listen to music (via headphones) during (es)ketamine administration was compared with those who prefer a setting without music. The aim was to gain exact information about the influencing factors of side effects of (es)ketamine as well as factors that might influence the response, in order to optimize the treatment.

## Methods

An independent ethics committee in Switzerland (EKNZ) approved the study protocol and only data of patients who provided written informed consent was used in the study.

### Study design

This was a single center, retrospective observational study analyzing data since 26^th^ of February 2021 (beginning of the ambulant therapy with (es)ketamine in the center of affective disorders at UPK Basel) until 3^rd^ of July 2023.

### Study population

**Figure 1:**
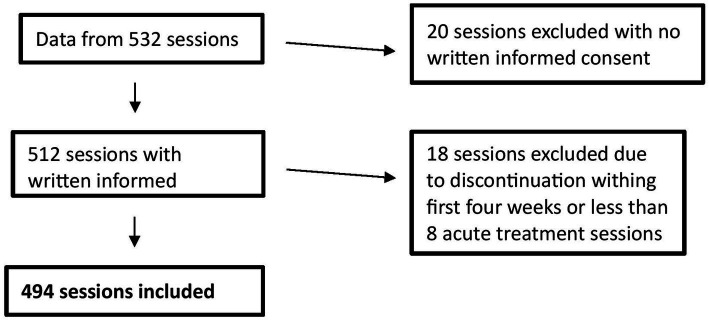
Flowchart of exclusion criteria.

Over the duration of the study, all patients were asked to sign a general consent of the hospital. Altogether, data of 45 patients with a diagnosis of MDD (Major Depressive Disorder), according to the International Classification of Diseases (ICD-10, 10th edition), based on the clinical examination and interview, entered the study. Adult patients receiving (es)ketamine intranasal therapy at the UPK for clinical reasons and who signed the written informed consent of the hospital were included. Patients with no written informed consent (one patient), discontinuation within first 4 weeks or less than 8 acute treatments (7 patients) were excluded. Since the comparison between the two groups (listening to music vs. not listening to music) was made on the basis of sessions rather than patients, 494 sessions were considered according to the exclusion criteria (Figure 1).

### Data protection

Source data for this study was obtained from electronic medical records. In the case of routinely collected source data, unencoded information was utilized, and each participant was assigned a unique study participant number (study-ID). The health-related patient data that was collected was then stored using electronic Case Report Forms (eCRF) through the REDCap® system. Within the eCRF, participants were exclusively identified by their unique participant number.

### Intranasal (es)ketamine administration and measurement protocol

Prior treatment with commercial esketamine, a cost approval is required according to a strict procedure. Following points must apply for the basic insurance in Switzerland to cover the costs: At least two antidepressants must have failed over a sufficient time and dosage, augmentation with lithium or atypical antipsychotic in the past, electroconvulsive therapy not possible or refused and current use of an oral antidepressant. Patients with TRD who do not qualify for intranasal esketamine treatment but agree to the off-label use of intranasal therapy with (es)ketamine receive racemic ketamine. The use of racemic ketamine as a nasal spray has been shown to be safe and effective in the treatment of depressive symptoms in real-world sample of patients hospitalized with TRD (36) and significantly cheaper than commercially available esketamine (37). The treatment protocol requires regular assessment of blood pressure, dissociation and severity of depressive symptoms as well as (es)ketamine-dose and setting characteristics, such as listening to music (the patient decides whether or not to listen to music during the session).

Both esketamine and racemic ketamine were provided in a disposable device. Until now, only one esketamine drug was approved in Switzerland, containing a total dose of 28 mg esketamine per device with two strokes. The racemic ketamine is produced by the university hospital pharmacy and contains a solution of 100 μL and 14 mg racemic ketamine per stroke.

**Figure 2:**
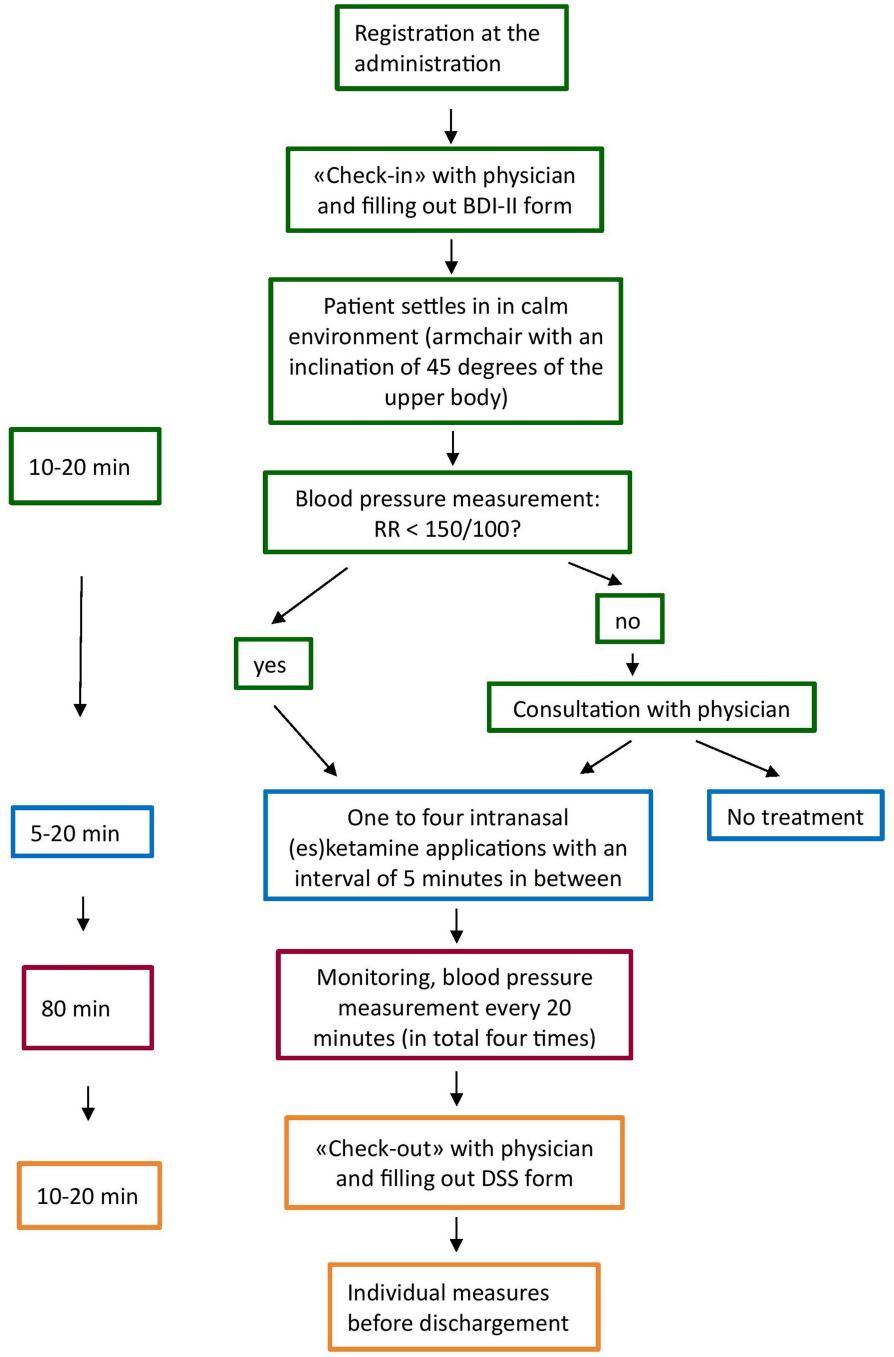
Procedure of intranasal (es)ketamine administration.

The treatment schedule at UPK Basel was as follows: For the first 4 weeks, patients receive an acute treatment, i.e., twice a week intranasal (es)ketamine, followed by the maintenance phase, which starts with one administration per week and then a gradually reduced frequency of administration. Figure 2 shows the procedure of the intranasal (es)ketamine application is depicted. After filling out the self-reported BDI-II form (Beck’s Depression Inventory) and seeing the physician for a check up on the general psychological wellbeing, a MADRS (Montgomery–Åsberg Depression Rating Scale) and a potential adjustment of the dosage, patients were placed in an inclinated position (45 degrees of the upper body) in a chair with armchair, in a quiet environment (one or two patients in the same room, separated by curtain). After an initial blood pressure measurement, (es)ketamine was administrated once up to four times (according to the dosage) with an interval of 5 min in between. All patients received instructions before the first administration. After application, each patient was monitored, including a blood pressure measurement every 20 min. In case of discomfort or side effects, the nurse informed the physician in charge to evaluate the situation and to take individual measures, if needed. After about 80 min, all patients filled out the dissociation scale (DSS-IV), anxiety and euphoria analogue scale and saw the physician before dischargement (Figure 2).

### Measurement forms

*BDI-II*: The self-reported Beck Depression Inventory-II is a revised version of the depression inventory developed by Aaron Beck and consists of 21 items. BDI-II items are rated on a 4-point scale ranging from 0 to 3 based on severity of each item. The maximum total score is 63. The German version demonstrated good reliability and validity in clinical and nonclinical samples (38).

*DSS-IV*: The self-reported Dissociation Symptoms Scale-IV is a valid instrument to easily assess psychological and somatic aspects of dissociative states. It consists of the following 4 items:

1. I have the impression that my body does not belong to me (depersonalization).
2. I have problems hearing, e.g., I hear sounds from nearby as if they come from far away (somatoform dissociation).
3. I have the impression other people or things around me are unreal (derealization).
4. I have the impression that my body or parts of it are insensitive to pain (analgesia).

Each item ranges from 0 to 10 based on the level of severity (39).

*MADRS*: The clinician-rated Montgomery–Åsberg Depression Rating Scale is designed to measure the severity of depression and has proven good reliability and validity in the original and German version (40). It contains the following 10 items: apparent sadness, reported sadness, inner tension, sleep, appetite, concentration, lassitude, inability to feel (interest level), pessimistic thoughts, and suicidal thoughts. Each item ranges from 0 (no symptom/normal) to 6 (severe/continuous symptom), with a total possible score of 60 (41).

### Statistical analysis

Statistical tests were conducted at a significance level of 0.05 and analyses were performed using R-Studio software (version 1.2).

First, a descriptive analysis has been performed to summarize the sample. Median and mean has been used as indicators of central tendency, range, and standard deviation as indicators of the variability. The normality of the distribution was assessed using the Shapiro–Wilk test. Variables have been considered as non-normally distributed if the *p*-value was less than α =0,05. Weight, height, BMI, initial BDI, initial MADRS, first systolic BP (blood pressure) of each patient, first diastolic BP of each patient and initial systolic BP from all the sessions met the normality assumption. Age, dose, initial diastolic BP form all the sessions, maximum systolic BP, maximum diastolic BP, DSS Item 1, DSS Item 2, DSS Item 3, DSS Item 4, anxiety, euphoria and pleasantness were non-normally distributed.

Additionally, a generalized additive mixed model was used to compare the two groups (music listening vs. no music) while considering that each patient had the choice to listen to music or not at the beginning of each therapy session. In this analysis, the units of observation were the individual therapy sessions rather than the patients themselves. Generalized additive models were selected to account for non-normal distribution (42). We utilized the R Package ‘mgcv., ‘Version 1.9–0. This approach was selected to address the challenge of repeated measurements and random effects arising from multiple assessments of the same patient. The model-building process consisted of two steps: In the first step, univariate models were constructed individually for each variable of interest. In the second step, multivariate models were developed using only the variables that demonstrated a *p*-value <0.05 in the first step. This approach was employed to address potential confounding factors. In general, a *p*-value <0.05 in the final model was considered statistically significant.

### Endpoints

The primary endpoints included the elevation in systolic blood pressure, elevation in diastolic blood pressure, assessment of dissociation using DSS-IV, evaluation of anxiety and euphoria through an analogue scale, and measurement of pleasantness. The secondary endpoint was the average dosage of (es)ketamine administered. These endpoints were evaluated for both sessions with and without listening to music.

## Results

### Study population

Out of the 37 patients, 6 (16%) had a bipolar affective disorder with a current depressive episode (F31.3–5) and 31 (84%) had a recurrent depressive disorder (F33). The overall mean of the initial BDI score was 30, for the main diagnoses 18.2 and 32.3, respectively. And the overall mean of the initial MADRS score was 23.6, for the main diagnoses 15.3 and 25.2, respectively (Table 1).

**Table 1:** Baseline chart: description of population and sessions.

*Population*			*Sessions*		
Characteristic	*N* = 37		Characteristic	*N* = 494	
	Mean (SD)	95% CI		Mean (SD)	95% CI
Sex			Acute or maintenance		
Female	24 (65%)		Acute	223 (45%)	
Male	13 (35%)		Maintenance	271 (55%)	
Age, yr	47.1 (10.8)	43.6, 50.6	Dose, mg	124.9 (42.4)	111.2, 138.6
BMI, kg/m^2^	17.4 (5.5)	15.6, 19.2	Initial syst. BP	130.0 (12.6)	125.9, 134.1
Smoking	13 (35%)		Max. syst. BP	138.7 (13.9)	134.2, 143.2
Systolic BP	130.6 (11.5)	126.3, 133.7	Increase syst. BP	8.6 (12.7)	4.5, 12.7
Diastolic BP	83.9 (9.5)	80.8, 87.0	Initial diast. BP	83.0 (10.6)	79.6, 86.4
Main diagnosis			Max. diast. BP	90.0 (9.8)	86.8, 93.2
F31.3–5 Bipolar affective disorder, current depressive episode	6 (16%)		Increase diast. BP	7.0 (8.9)	4.3, 9.9
F33 Recurrent depressive disorder	31 (84%)		DSS Item 1	2.7 (2.8)	1.8, 3.6
Initial BDI	30.0 (12.8)	25.9, 34.1	DSS Item 2	2.0 (2.5)	1.2, 2.8
Last BDI	21.7 (13.1)	17.5, 26.0	DSS Item 3	1.9 (2.6)	1.1, 2.7
Difference BDI	−8.8 (10.6)	−11.4, −4.6	DSS Item 4	2.5 (2.7)	1.6, 3.7
Initial MADRS	23.6 (11.1)	20.0, 27.2	Anxiety	0.8 (1.7)	0.3, 1.3
Last MADRS	16.8 (9.7)	13.7, 19.9	Euphoria	1.8 (2.5)	1.0, 2.6
Difference MADRS	−6.8 (10.8)	−10.3, −3.3	Pleasantness	6.1 (2.0)	5.5, 6.7
Sessions	14 (6.3)	11.7, 15.8	Listening to music		
			Music	234 (52%)	
			No music	216 (48%)	

The average age of this population was 47 and the average BMI was 27.4. 24 (65%) patients were female and 13 (35%) patients were male. 13 (35%) were smoking with a mean of 24.7 pack years. For the average blood pressure, the initial measurement of the first session before the (es)ketamine application of each patient was taken to avoid bias, as the number of sessions per patient varies greatly. The mean systolic blood pressure was 130.6 mmHg and the mean diastolic blood pressure was 83.9 mmHg.

Psychiatric comorbidities were attention deficit hyperactivity disorder, alcohol dependency (both in four cases), post-traumatic stress disorder, mixed personality disorder, borderline personality disorder (all in three cases), obsessive compulsive disorder, abuse of hypnotics (in two cases), dysthymia, attention deficit disorder, generalized anxiety disorder, panic disorder, social phobia, narcistic personality disorder, dysmorphic disorder, bulimia, autism and somatoform disorder (in one case).

Somatic comorbidities included obesity, arterial hypertension (both in 5 cases), hypothyroidism (3), atrial fibrillation, diabetes mellitus (both in two cases), supraventricular tachycardia, ulcerative colitis, Morbus Crohn, Morbus Bechterew, back pain syndrome, acne inverse, Lupus erythematodes tumidus, neurodermatitis, epilepsy, long covid and somatoform pain disorder (all in one case).

**Figure 3:**
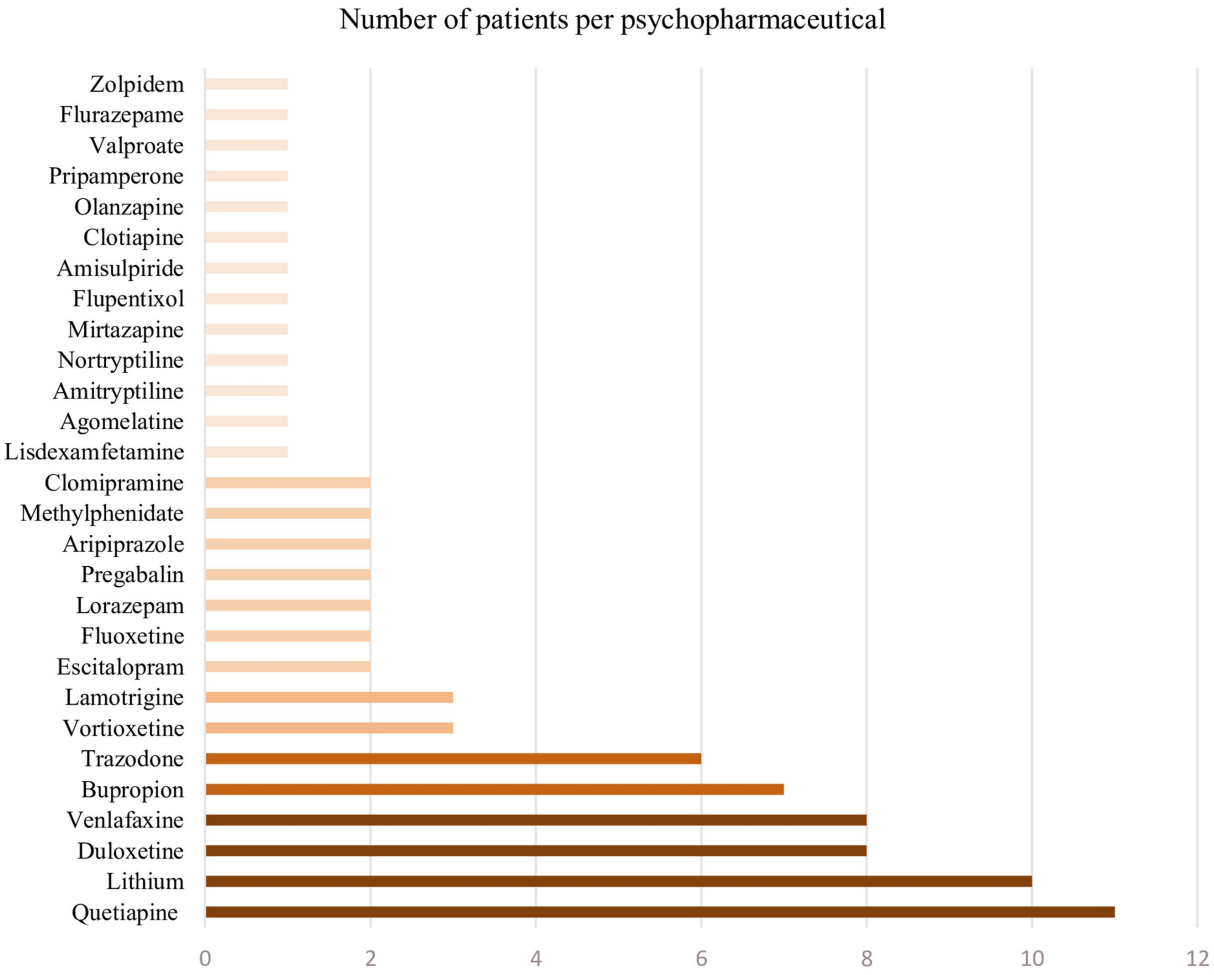
Psychopharmaceuticals.

Psychopharmaceuticals taken are summarized in Figure 3.

Other medications consisted of levothyroxine (3), levothyroxine (3), pantoprazol (2), rivaroxaban, ramipril, candesartan, amlodipin, bisoprolol, propranolol, torasemid, ramipril, esomeprazol, insulin, metformin, liraglutid, tizanidin, dafalgan and chondroitin (all in once case).

All patients were in ambulatory psychiatric treatment, mostly by the person who referred them. For 34 (92%) of the patients it was the first time receiving an intranasal (es)ketamine therapy. 31 (84%) patients received the racemic ketamine and 6 (16%) patients received esketamine. The mean (95% CI) of sessions per patient was 14 (11.7, 15.8).

The mean of the initial BDI score was 30.0 and the mean of the last BDI score was 21.7, making it an average improvement of 8.8 points. And the mean of the initial MADRS score was 24 and the mean of the last MADRS was 16.8, making it an average improvement of 6.8 points. Remission is defined as a MADRS total score ≤ 12, which is true for 16 (43%) patients. The response rate is defined as ≥50% reduction in total MADRS score from baseline, which is true for 11 (30%) patients. The partial response is defined as a reduction between 50 and 0%, which is true for 17 (46%) patients. For the other 9 (24%) patients, there was no response.

### Sessions

In total, 494 sessions in the period from the 26th of February 2021 until the 3rd of July 2023 were included in the study. 223 (45%) were acute and 271 (55%) were maintenance treatment. The mean dose was 124.9 mg for all the sessions. The difference between the initial blood pressure (systolic mean of 130.0 mmHg and diastolic mean of 83.0 mmHg) and the highest following blood pressure (systolic mean of 138.7 mmHg and diastolic mean of 90.0 mmHg) was evaluated. There was an increase of the blood pressure after the administration of (es)ketamine, an average of 8.6 mmHg for the systolic and an average of 7.0 mmHg for the diastolic blood pressure (Table 1). Medically relevant side effects that required intervention, occurred in 10 cases (2% of all sessions): nausea (5), emesis (2) and symptomatic hypertension (3).

During the sessions, patients could listen to their own music via headphones. So, there was no pre-prepared playlist. Nevertheless, the patients were asked about their choice of music. Answers included music genres such as meditation music (3), relaxing music (3), instrumental music (2), classical music (2), pop ballads (2), Indian music (1) and popular rock and pop bands. In summary, the music selection was often without lyrics, plainly instrumental and rather subjectively relaxing, than exciting. However, the specific choice of music genre was not systematically assessed since it was not a primary objective. In total, patients listened to music during 234 (52%) sessions, while no music was listened to during the other 216 (48%) sessions. In 44 sessions, it was not stated whether music was listened to or not. 17 (46%) patients listened mostly (more than 50% of sessions) to music and the other 20 (54%) patients did not listen to music most of the time.

Since listening to music was not fixed for the entire duration of each patient’s therapy, but each patient could decide at the beginning of the session whether they wanted to listen to music or not, the comparison between the two groups was made on the basis of sessions and not on the basis of patients. For example, the change in the BDI score and the change in the MADRS score between the beginning and end of a patient’s therapy were not used for comparison, but the change values between successive sessions.

### Comparison between the two groups

In univariate comparison of the two groups (listening to music vs. not listening to music), there was a significant difference in the variables dose (*p*-value: <0.001, β: 13.2), Max. syst. BP (*p*-value: 0.011, β: -3.1), DSS Item 1 (*p*-value: 0.018, β: 0.6), DSS Item 4 (*p*-value: 0.046, β: 0.5), anxiety (value of p: <0.001, β: −1.0) and pleasantness (*p*-value: <0.001, β: 0.7) (Table 2).

**Table 2:** Univariate comparison of the two groups (listening to music vs. not listening to music).

Characteristic	Music, *N* = 234		No Music, *N* = 216		*p*-value^I^	β^II^	R^2^
	Mean (SD)	95% CI	Mean (SD)	95% CI			
Acute or maintenance							
Acute	96 (41%)		112 (52%)				
Maintenance	138 (59%)		104 (48%)				
**Dose, mg**	**131.5 (42.9)**	**126.0, 137.0**	**116.7 (40.5)**	**111.3, 122**	<0.001***	**13.2 (3.8)**	**0.104**
BDI change score	0.1 (6.0)	−0.7, 0.9	0.0 (5.3)	−0.7, 0.7	0.9		
MADRS change score	0.0 (7.1)	−0.9, 0.9	−0.5 (6.3)	−1.3, 0.3	0.4		
Initial syst. BP	129.3 (12.6)	127.7, 130.9	131.0 (12.5)	129.3, 132.7	0.2		
**Max. syst. BP**	**137.9 (12.6)**	**136.3, 139.5**	**140.3 (15.5)**	**138.2, 142.4**	0.011*	**−3.1 (1.2)**	**0.154**
Increase syst. BP	8.5 (12.4)	6.9, 10.1	9.3 (13.2)	7.5, 11.1	0.3		
Initial diast. BP	82.7 (11.7)	81.2, 84.2	83.4 (9.7)	82.1, 84.7	0.4		
Max. diast. BP	90.0 (9.7)	88.8, 91.2	90.2 (10.5)	88.8, 91.6	0.9		
Increase diast. BP	7.3 (9.9)	6.0, 8.6	6.8 (8.1)	5.7, 7.9	0.6		
**DSS Item 1**	**3.0 (3.0)**	**2.6, 3.4**	**2.4 (2.6)**	**2.1, 2.7**	0.018*	**0.6 (0.2)**	**0.016**
DSS Item 2	2.2 (2.7)	1.9, 2.5	1.9 (2.4)	1.6, 2.2	0.2		
DSS Item 3	2.1 (2.5)	1.8, 2.4	1.7 (2.6)	1.4, 2.0	0.2		
**DSS Item 4**	**2.8 (2.8)**	**2.4, 3.2**	**2.2 (2.6)**	**1.9, 2.5**	0.046*	**0.5 (0.3)**	**0.018**
**Anxiety**	**0.4 (1.1)**	**0.3, 0.5**	**1.4 (2.1)**	**1.1, 1.7**	<0.001***	**−1.0 (0.2)**	**0.104**
Euphoria	1.7 (2.5)	1.4, 2.0	1.9 (2.6)	1.6, 2.2	0.3		
**Pleasantness**	**6.4 (1.9)**	**6.2, 6.6**	**5.8 (1.9)**	**5.5, 6.1**	<0.001***	**0.7 (0.2)**	**0.046**

I Generalized additive mixed models. II Regression coefficient (SE). ^*^p <0.05; ^**^p <0.01; ^***^p <0.001. The bold values are statistically significant.

When comparing the two groups (listening to music vs. not listening to music) in a multivariate modell, there was only a significant difference in the variables dose (*p*-value: 0.003, β: 0.002), Max. syst. BP (*p*-value: 0.011, β: -0.004), DSS Item 1 (*p*-value: 0.005, β: 0.041) and anxiety (*p*-value: <0.001, β: −0.093). The value for the adjusted R square is 0.15 (Table 3). The dose and the above mentioned significant variables did not correlate with each other and anxiety correlated negatively only with DSS Item 3 and DSS Item 4, for which a multivariate mixed model was also used.

**Table 3:** Multivariate comparison of the two groups (listening to music vs. not listening to music).

Characteristic	Music, *N* = 234		No Music, *N* = 216		*p*-value^I^	β^II^
	Mean (SD)	95% CI	Mean (SD)	95% CI		
**Dose, mg**	**131.5 (42.9)**	**126.0, 137.0**	**116.7 (40.5)**	**111.3, 122**	<0.003**	**0.002 (0.001)**
**Max. syst. BP**	**137.9 (12.6)**	**136.3, 139.5**	**140.3 (15.5)**	**138.2, 142.4**	0.017*	**−0.004 (0.002)**
**DSS Item 1**	**3.0 (3.0)**	**2.6, 3.4**	**2.4 (2.6)**	**2.1, 2.7**	0.005**	**0.041 (0.013)**
DSS Item 4	2.8 (2.8)	2.4, 3.2	2.2 (2.6)	1.9, 2.5	0.492	
**Anxiety**	**0.4 (1.1)**	**0.3, 0.5**	**1.4 (2.1)**	**1.1, 1.7**	<0.001***	**−0.093 (0.015)**
Pleasantness	6.4 (1.9)	6.2, 6.6	5.8 (1.9)	5.5, 6.1	<0.3	

I Generalized additive mixed models. II Regression coefficient (SE). ^*^p <0.05; ^**^p <0.01; ^***^p <0.001. The bold values are statistically significant.

### Discussion

The main objective of this primary explorative study was to examine the influence of listening to music during intranasal (es)ketamine administration on tolerability and efficacy. Two groups (sessions with listening to music and sessions without listening to music) were compared.

No impact of listening to music during therapy session on the efficacy of (es)ketamine was observed, as assessed by changes in MADRS score and BDI score. It should be emphasized that the change in MADRS score and BDI score was evaluated from consecutive sessions, i.e., from one session to the next and not from the beginning to the end of a patient’s therapy.

While effectiveness remained unchanged, following effects of listening to music on side-effects and tolerability of (es)ketamine could be demonstrated.

First, the degree of dissociation was increased in the group listening to music, namely the DSS Item 1 (mean difference of +0.6 points). DSS Item 2, DSS Item 3 and DSS Item 4 were not significantly different in the two groups. Listening to music during (es)ketamine seems to cause more depersonalization (DSS Item 1), rather than visual dissociation (DSS Item 2), derealization (DSS Item 3) and analgesia (DSS Item 4). In line with previus findings, we assume the increased tolerability while listening to music is independent of the level of dissociation (15, 22). Furthermore, our findings regarding higher score in depersonalization in sessions with listening to music should be discussed in the context on the current debate if and in which extend dissociative symptoms correlate with effectiveness of (es)ketamine treatment (43). While some studies demonstrated association between dissociative symptoms and effectiveness of (es)ketamine (44–46), there are also opposite findings (47, 48). Niciu et al. hypothesize a potential causal link between specific dissociative feature (depersonalization) and antidepressant response initiated by glutamate modulation (45). Also findings of Hack et al. (49) suggest a prominent role of specific dissociative symptoms like depersonalization. Interestingly, depersonalization was the only dissociative phenomena, which positively correlated with listening to music in the present study. This finding supports the importance of the assessment of distinct dissociative symptoms, when tolerability and effectiveness are considered. Second, in the group listening to music the level of anxiety was decreased (mean difference of −1 point) and the sessions were perceived as more pleasant (mean difference of +0.6 points). Interestingly and contrary to our hypothesis a higher level of dissociation does not automatically go hand in hand with a higher level of anxiety, although there is still a correlation to be found. Previous studies showed, that listening to music significantly improves the tolerance to dissociative symptoms (15) and thereby reducing associated symptoms, such as distress, confusion, agitation and anxiety (22). This also applies to dissociative symptoms after (es)ketamine anaesthesia, where music improves the acceptance of these symptoms (21). Although intranasal (es)ketamine at the doses used does not cause high entropy states in the brain, dissociative symptoms can still occur. Here, the use of music in a safe and comfortable environment can help maintain a neutral or positive emotional tone (34) and let go of negative appraisals about these dissociative symptoms (15).

Third, the maximal systolic blood pressure after administration of es(ketamine) was significantly lower in the group listening to music, while there was no significant difference between the two groups in initial blood pressure measurements. These findings are supported by previous studies, where music therapy was an effective intervention for reducing blood pressure in individuals with hypertension (50). Since elevations in blood pressure can occur when (es)ketamine is administrated, listening to music could be a cost-effecitve und easy way to reduce this adverse effect.

Fourth, the dose in the group listening to music is significantly higher (mean difference of 14.8 mg). During the titration phase, the dose was increased if well tolerated and decreased if poorly tolerated or if response could be observed. Current findings suggest that adverse effects are dose-related (5). Since the group that listened to music showed decreased levels of anxiety, we hypothized, that listening to music increases subjective tolerability, which leads to (objective) higher tolerability of (higher) (es)ketamine doses. Whether or not the subjective tolerability differs between the groups (listening to music vs. not listening to music) can neither be confirmed nor denied, since only the above-mentioned parameters (dissociation, anxiety and pleasantness) were quantitively assessed.

Following limitations of this study should be mentioned. First, the influence of listening to music on efficacy is limited due to the study design (groups were identified by self-choice). For better evaluation a randomized controlled trial is needed. Second, not all side effects were recorded with visualization scales. Apart from dissociation, anxiety and well-being, there was no systematic quantitative recording of other side effects (nausea, dizziness, sedation, motoric symptoms). The assessed 2% of cases requiring intervention were not divided between the two groups due to the low total number (10).

Third, the music selection was made by the patients themselves. More data is needed to evaluate the influence of specific music genres. However, the focus of this study was less on the specific choice of music but on the influence of music which was chosen by the patients themselves. Previous research indicates that self-selected music is as effective as predetermined music to reduce preoperative anxiety in gynaecological interventions (51) and that listening to music selected by the patients themselves reduced preoperative anxiety (52). Another study showed, that the source of relaxation is not necessarily a particular genre of music but rather the perception or belief that the music is calming or soothing (53). Nevertheless, there was a trend to choose music without lyrics and designed for relaxation, which is in line with a study showing that soothing music has a positive effect on relaxation in Taiwanese elderly people (54).

In summary, these findings provide valuable insights into the management of side effects during intranasal therapy with (es)ketamine, offering opportunities for treatment optimization in the future. Music, as one of the supportive interventions, presents a straightforward and cost-effective approach to enhancing the treatment environment. It aids in improving the tolerance of potential dissociation, reducing associated anxiety, reducing blood pressure and facilitating the acceptance of higher doses. These findings highlight the potential for a holistic approach to treatment that considers patient comfort and well-being alongside therapeutic efficacy, ultimately contributing to more effective and patient-centered care.

## Data availability statement

The raw data supporting the conclusions of this article will be made available by the authors, without undue reservation.

## Ethics statement

The studies involving humans were approved by Ethikkommission Nordwestschweiz. The studies were conducted in accordance with the local legislation and institutional requirements. The participants provided their written informed consent to participate in this study.

## Author contributions

JH: Data curation, Formal analysis, Visualization, Writing – original draft, Writing – review & editing. JS: Conceptualization, Methodology, Supervision, Writing – original draft, Writing – review & editing. TL: Data curation, Methodology, Supervision, Writing – review & editing. AB: Resources, Supervision, Writing – review & editing. UL: Conceptualization, Resources, Supervision, Writing – review & editing.
